# A Tablet Computer–Based Food Record for the Self-Assessment of Nutritional Intake in Patients Undergoing Geriatric Rehabilitation: Prospective Pilot Feasibility Study

**DOI:** 10.2196/84653

**Published:** 2026-07-09

**Authors:** Julia Berndt, Patrick Elfert, Marco Eichelberg, Juergen Martin Bauer, Andreas Hein, Rebecca Diekmann

**Affiliations:** 1 Assistance Systems and Medical Device Technology, Department of Health Services Research, Carl von Ossietzky University, Oldenburg, Germany; 2 Nutrition and Physical Function in Older Adults, Department of Health Services Research, Carl von Ossietzky University, Ammerländer Heerstraße 140, Oldenburg, 26129, Germany, 49 441798 ext 4359; 3 OFFIS-Institute for Information Technology, Oldenburg, Germany; 4 Center for Geriatric Medicine, Agaplesion Bethanien Hospital, Heidelberg, Germany

**Keywords:** eHealth, nutrition, geriatric rehabilitation, tablet computer, food record, older adults, technology, nutrition app, feasibility study

## Abstract

**Background:**

Nutritional status is an influential factor for functional status and rehabilitation outcomes in patients undergoing geriatric rehabilitation. Although there is evidence for the potential of eHealth interventions in patients undergoing geriatric rehabilitation in general, the evidence for eHealth interventions with a focus on nutrition is scarce. In other target groups with older people, eHealth applications to support nutrition, such as computer-based food records, have been used successfully.

**Objective:**

Therefore, the aim of this study was to verify whether it is feasible for patients undergoing geriatric rehabilitation to independently use a tablet computer−based food record (e-food record) to document their food and beverage intake. The e-food record was developed in advance and tailored to the age- and disease-specific needs of patients undergoing geriatric rehabilitation.

**Methods:**

This prospective pilot study investigated the general feasibility of an e-food record in older adults (≥70 y) in a geriatric rehabilitation center in Germany. It was tested whether the e-food record could be independently used by the participants over 3 days. Furthermore, the usability of the e-food record was assessed by the System Usability Scale (0‐100 points) after usage. To compare nutritional data, the participants recorded their consumption of food and beverages by the e-food record and by a 24-hour recall for the same time period, and the mean difference was calculated as follows: the value of the 24-hour recall minus the value of the e-food record. As the study was characterized as a pilot, the data analysis was descriptive.

**Results:**

Seventeen out of 25 patients (n=6, 35.3% female, mean age 79.5, SD 3.7 y) maintained the e-food record independently over the study period. The mean System Usability Scale score of the e-food record was 76.0 (SD 11.3) points. Datasets of 9 out of 17 participants (n=5, 55.6% female, mean age 78.2, SD 2.9 y) were analyzed in terms of nutritional data. Mean differences in energy, protein, and fluid intake by the 24-hour recall compared to the e-food record were 4.9 (SD 10.2) kcal/kg body weight (bw), 0.1 (SD 0.3) g/kg bw, and 4.9 (SD 9.4) g/kg bw, respectively.

**Conclusions:**

The use of an e-food record is generally feasible for patients undergoing geriatric rehabilitation characterized by low technical experience, high mean age, and a high rate of functional impairment. Lower intake levels were observed for the e-food record compared to the 24-hour recall with regard to energy, protein, and fluid intake. Aspects for further development of the e-food record were identified to enable evaluation on a larger sample. Following successful evaluation, the e-food record could be used within nutrition therapy in the future to increase the efficiency of the nutritional therapy process.

## Introduction

### Background

Older people represent a growing population in Western countries due to sociodemographic developments. As a consequence, the number of people who have therapeutic or nursing needs is also increasing sharply [1]. Therefore, geriatric rehabilitation is becoming increasingly important in the medical care of older people. Patients undergoing geriatric rehabilitation are characterized by a reduced functional status due to disabling impairments. These are often combined with the presence of multimorbidity and geriatric syndromes such as sarcopenia or frailty [2-4]. The aim of geriatric rehabilitation is to improve functional resources in order to enable patients to return to their home environments [5].

Nutritional status is a well-known factor that is interrelated with physical function, frailty, and sarcopenia, which are associated with negative effects on functional dependence in patients undergoing geriatric rehabilitation [6-8]. However, malnutrition and the risk of malnutrition are widespread conditions among these patients, with a prevalence of 5% to 20% for malnutrition and 40% to 54% for the risk of malnutrition [8]. Because there is profound evidence, malnutrition is treated as part of the structured rehabilitation program within nutritional therapy [9]. The focus here is on optimizing energy and protein intake and on weight stabilization to generate a positive effect on muscle building and muscle maintenance [10]. The amount of energy and protein intake consumed is determined by the nutritional therapist using the method of a food record. Individual nutritional recommendations for the patient are then derived and communicated on this basis. Due to financial and personnel resources, nutritional therapy is often limited in time during rehabilitation treatment, even if the literature indicates that close-meshed nutritional therapy might achieve greater success [11].

eHealth applications, such as computer-based food records, can be used to provide support by intensifying the nutrition therapy process. The time-consuming processes of recording and analyzing dietary intake data are no longer necessary for the nutrition therapist. Maintaining a computer-based food record regularly enables the diet to be monitored more consistently and proactively. Deviations from the nutrition target can be recognized earlier, and reintervention can be initiated by the nutrition therapist. In addition, maintaining the computer-based food record can increase the patient’s awareness of their diet and therefore might lead to higher motivation in achieving nutritional goals. In the context of geriatric rehabilitation, advanced age, limited experience with technology, reduced visual acuity, changes in haptics, cognitive limitations, and fears in dealing with technology are characteristic challenges when using eHealth systems in this target group that must be addressed in the technical development process.

eHealth interventions in geriatric rehabilitation have already been shown to have the potential to improve rehabilitation outcomes. In a meta-analysis, the evidence for feasibility, usability, and effectiveness in the treatment of patients undergoing geriatric rehabilitation was demonstrated. However, the eHealth interventions focused predominantly on activity and physical function. No nutritional interventions were reported [12]. Only Happe et al [13,14] demonstrated in 2022 and 2023 the feasibility and usability of a tablet-based e-coach to improve nutrition and physical activity in patients undergoing geriatric rehabilitation. As far as we know, there is no research data on e-food record methods in the group of patients undergoing geriatric rehabilitation published to date.

### Objectives

Therefore, the primary objective of this pilot feasibility study was to verify whether it is fundamentally possible for patients undergoing geriatric rehabilitation with low technical experience and simultaneous high postacute or chronic stress to independently use a tablet computer−based food record (e-food record) over 3 days that has been specially adapted to their needs. Secondary and exploratory objectives included the assessment of usability as well as the analysis of the recorded dietary data. In addition, the recorded food items, food portions, and nutritional intake data of the e-food record are compared to the recorded data of a 24-hour dietary recall, a commonly used reference method in clinical practice. Finally, the study aimed to identify potential areas of improvement to inform further development of the e-food record.

## Methods

### Participants and Study Design

Patients aged 70 years and older who were consecutively admitted to a geriatric rehabilitation ward of a rehabilitation center in the northwest of Germany were eligible for the present prospective pilot feasibility study. Exclusion criteria were (1) immobility in the sense of confinement to bed, (2) the inability to understand the study content and the course of the study, (3) difficulties in communication in the German language, and (4) participation in another study. Inpatient geriatric rehabilitation in Germany is a structured multidisciplinary program with the goal of regaining independence and functionality in older persons with reduced functional conditions due to acute or chronic medical conditions [15]. Daily therapy is administered for a period of usually 20 days [16], including medical therapy, nursing care, physiotherapy, occupational therapy, neuropsychology and social services, nutritional therapy, and speech therapy [15]. Patients undergoing geriatric rehabilitation represent a highly vulnerable and clinically burdened population, often characterized by multimorbidity, functional limitations, and increased physical and psychological stress following acute or chronic health events. Furthermore, familiarity with and affinity for digital technologies are often limited in this age group. Data for the feasibility study were collected between July and December 2018. All participants took part in the study during the geriatric rehabilitation program. Sex and age were requested from the patients and documented. Due to ethical and data protection regulations, no data were collected from patients who declined participation. Therefore, no systematic information on reasons for nonparticipation is available.

### Ethical Considerations

The study was approved by the medical ethics committee of the Carl von Ossietzky Universität Oldenburg (ethical approval code 2018-029). We conducted the study in accordance with the Declaration of Helsinki. The study was a priori registered in the German Clinical Trials Register (DRKS-ID: DRKS 00014370).

Informed consent was obtained from all participants prior to participation. Pseudonymized data were collected and stored on secure, password-protected systems accessible only to the research team. Results are reported in aggregated or anonymized form. Participants did not receive any financial compensation, incentives, or other benefits for their participation in this study.

### Study Assessment of Food Records

#### e-Food Record

**Figure 1.**
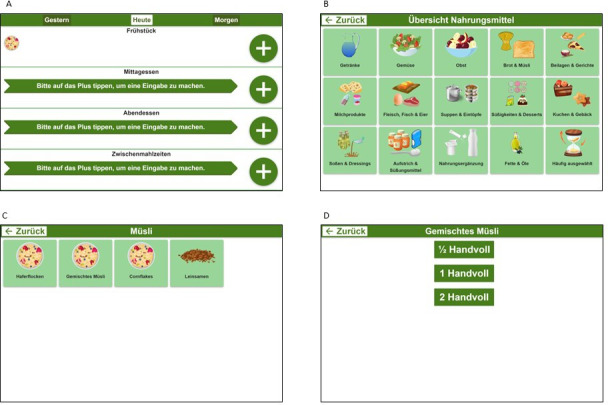
Screen views of the e-food record in the original German version according to Elfert [20]. (A) Start screen with an entry of muesli under breakfast and the prompt to make an entry for lunch, dinner, or snacks by tapping on the plus symbol. (B) Screen view for the selection of a food upper group (eg, beverages, fruits, milk products, soup and stew, and fat and oil). (C) Screen view for food selection using the example of muesli. (D) Screen view for portion selection using the example of muesli. English translations are as follows: Gestern: yesterday; Heute: today; Morgen: tomorrow; Frühstück: breakfast; Mittagessen: lunch; Abendessen: dinner; Zwischenmahlzeiten: snacks; Bitte auf das Plus tippen, um eine Eingabe zu machen: Please tap the plus sign to enter an entry; Zurück: back; Übersicht Nahrungsmittel: food overview; Getränke: beverages; Gemüse: vegetables; Obst: fruits; Brot & Müsli: bread & cereals; Beilagen & Gerichte: side dishes; Milchprodukte: dairy products; Fleisch, Fisch & Eier: meat, fish & eggs; Suppen & Eintöpfe: soup & stew; Süßigkeiten & Desserts: sweets; Kuchen & Gebäck: cakes; Soßen & Dressings: sauces; Aufstrich & Süßungsmittel: spreads & sweeteners; Nahrungsergänzung: supplements; Fette & Öle: fat & oil; Häufig ausgewählt: frequently selected; Müsli: cereals; Haferflocken: oatmeal; Gemischtes Müsli: mixed granola; Cornflakes: cornflakes; Leinsamen: flaxseeds; Handvoll: handful.

The patients’ food and beverage consumption was recorded by the patients themselves using an e-food record for 3 days including 2 weekdays and 1 weekend day. The e-food record used was an app-based computer program developed in close cooperation between experts in the field of nutritional counseling and app developers. It was installed on a 10.1-inch tablet Lenovo YOGA Tab 3 Plus in landscape mode. With regard to data protection, all data collected via the e-food record were stored locally on the device and transferred via secure local (offline) data transfer to a university computer after study completion. No cloud-based storage or external data transfer was used. No personal identifying information was stored within the application, and the access to study data was restricted to authorized members of the research team only. Technological aspects and the user-centered development of the e-food record were published by Elfert et al [17,18]. Design guidelines for apps for older people were key components in the development of the e-food record. The start screen of the e-food record was present at the current date and prompted the patients to make an entry for either breakfast, lunch, dinner, or a snack by touching the meal-associated plus signs. In total, 195 food symbols out of 14 food categories (eg, “bread and cereals,” “vegetables,” “meat, fish, and eggs,” and “beverages”) and 18 subcategories (eg, “bread,” “buns,” and “cereal” within the upper category “bread and cereals”) could be selected by the patients. The view of the start screen and the selection screen of the food upper categories are shown in Figure 1.

Portions suitable for everyday use were predefined in the e-food record based on the food pyramid of the Federal Center for Nutrition [19] and could be selected as half, 1, or 2 portions. Where possible, the portion sizes referred to an easily manageable hand measure. For example, one portion of bread equaled one slice of bread about the size of the hand, and one portion of cereals, fruits, vegetables, and sweets equaled one handful. Beverages were measured in glasses, cups, or mugs. There was no option for free entries. Before the application test, the participants were given a detailed introduction to the e-food record. For this purpose, they entered a defined sample meal into the e-food record under guidance, whereby open questions regarding the handling were addressed and clarified. All data were stored locally on the tablet computer and downloaded from the device via a cable connection upon successful completion of the study.

#### 24-Hour Recall—Paper-Based Food Record

In addition to the e-food record, a 24-hour recall was conducted retrospectively for each day the e-food record was also filled out. For this purpose, a prestructured paper-based template of a 24-hour recall was used to enter all consumed food and beverages of the previous day asked by a nutritionist and a member of the study staff. The prestructured 24 hour recall used here consisted of 18 food categories such as “bread,” “vegetables, salad,” “meat, fish, eggs,” “juices, water, other beverages,” etc. Each upper category contained 4 to 11 single foods. Predefined portions were counted as a tally for each of the 3 days. Food not listed was entered as free input at the end of the 24-hour recall.

#### Feasibility

Feasibility, defined as the independent use of the e-food record over 3 days, was assessed during a 3-day application test in inpatient geriatric rehabilitation. Feasibility was operationalized by the completion of the recording period and quantified using the dropout rate.

#### Usability and Technology Commitment

As secondary outcomes, usability and technology commitment were assessed. Following the use of the e-food record, the user experience was reflected upon. Usability was measured using the System Usability Scale (SUS, 0‐100 points) [21] in its German translation [22], with higher scores indicating better usability. The SUS is a standardized questionnaire with 10 items that are rated on a Likert scale from 1 to 5. A value of over 68 is considered above average. The SUS scores determined were grouped together and classified using adjectives according to Bangor et al [23] as follows: best imaginable (100-84.1 points), excellent (84-80.8 points), good (80.7-71.1 points), okay (71-51.7 points), poor (51.6-25.1 points), and worst imaginable (≤25 points).

Prior to the application test, the technology commitment was assessed according to Neyer et al, with a standardized 12-item questionnaire (12‐60 points). The items were rated on a 5-point Likert scale and reflect the areas of personal contact, interest, and expectations for the use of technologies [24].

### Exploratory Analysis: Comparison of E-Food Record and 24-Hour Recall

#### Nutritional Data

As an exploratory objective, dietary data obtained from the e-food record were compared with those from a 24-hour recall. The nutritional values of energy, protein, and fluid intake of the e-food record dataset and the 24-hour recall were adjusted for body weight (bw) and calculated as grams (g) divided by bw (kg) in a comparable manner for both protocol methods on the basis of the German Federal Food Code. Fluid intake was expressed in g/kg bw and not in mL/kg bw, as provided by the nutritional analysis software DGE Expert (version 1.3.14.1, Deutsche Gesellschaft für Ernährung) used in this study. The mean differences of energy, protein, and fluid intake between the e-food record and 24-hour recall were calculated by subtracting the values of the e-food record from the 24-hour recall, respectively. In addition, agreement in recorded food and beverage entries and portion sizes between both methods was explored. For this purpose, entries were grouped into predefined food categories.

#### Food and Beverage Portion Entries

After the 3-day application test, the entries of the recorded food and beverages along with the corresponding portion entries from the e-food record and the 24-hour recall were extracted. The recorded data of the 2 methods were grouped into the upper food categories of “beverages,” “vegetables,” “fruits,” “meat, fish, and eggs,” “dairy products,” “soup and stew,” “side dishes,” “sauces,” “sweets,” “cakes,” “bread and cereals,” “spreads,” “fat and oil,” “sweeteners,” and “supplements,” respectively.

### Statistical Analysis

The statistical analysis was conducted using SPSS Statistics version 29 (IBM SPSS Inc). Descriptive statistics are presented as mean values and SD, median and IQR, or as total numbers and percentages. Due to the pilot characteristics of the study, the statistical analysis refers to an explorative analysis, and no interference statistics were performed.

## Results

### Feasibility

**Figure 2.**
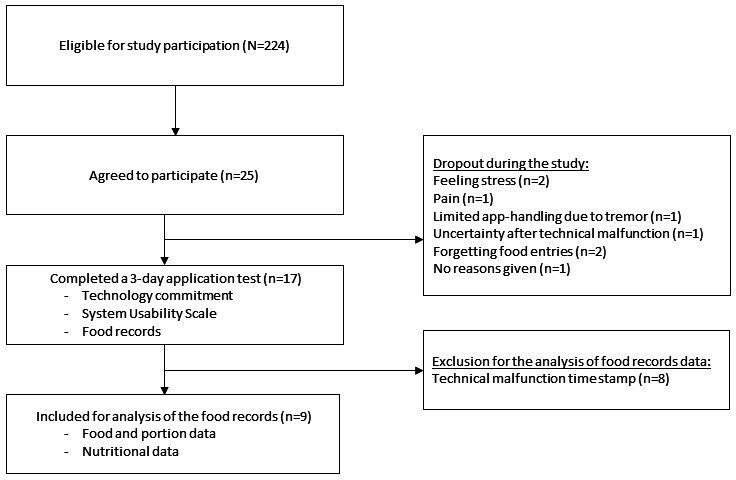
Flowchart for inclusion and dropout of study participants.

Twenty-five out of 224 (11%) patients who were eligible for inclusion were enrolled in the study. Seventeen out of 25 (68 %) patients completed the study and were included in the analysis of usability and technology commitment, while 8 patients dropped out of the study prematurely. Detailed information about the reasons for dropout is shown in Figure 2. During the study, a technical malfunction occurred in which the day selection did not automatically jump to the current day, contrary to what the study participants were told. As a result, the entries could no longer be validly assigned to the individual protocol days. Due to this, 9 out of 17 datasets were included in the analysis of the nutritional data (group A), while 8 datasets could not be analyzed (group B).

### Participant Characteristics

The mean age of all participants (n=17) who completed the study was 79.5 (SD 3.7) years. Six (35.3%) of them were female. The mean age of the participants who completed the 3-day application test (group A, n=9) was 78.2 (SD 2.9) years. Five out of 9 (55.6%) participants were female. The average age of the participants whose data were included for the analysis of the food records (group B, n=8) was 81.0 (SD 4.0) years, and 1 (12.5%) participant was female.

### Usability and Technology Commitment

The SUS was rated 76.0 (SD 11.3) points on average by all participants. It was higher in participants of group A with a mean score of 79.4 (SD 10.0) points compared to 72.2 (SD 11.4) points in participants of group B.

**Figure 3.**
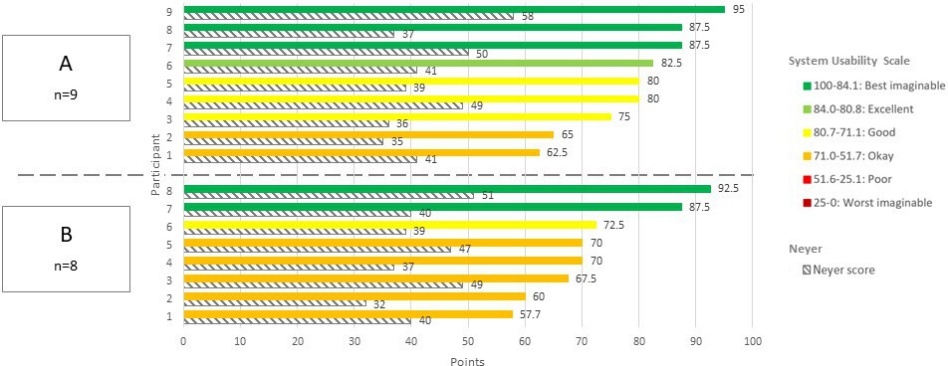
Usability and technology commitment per participant. Adjectives were used according to Bangor et al [23].

The mean technology commitment of all participants was 42.4 (SD 6.8) points. For group A, the mean technology commitment was 42.9 (SD 7.3) points compared to group B with 41.9 (SD 6.1) points. The single-item responses of the Likert scale (1‐5 points) were rated on average with 3.5 (SD 1.8) points (n=17). Detailed information on the values of the SUS and technology commitment per participant are represented in Figure 3.

### Comparison of e-Food Record and 24-Hour Recall

#### Nutritional Data

**Figure 4.**
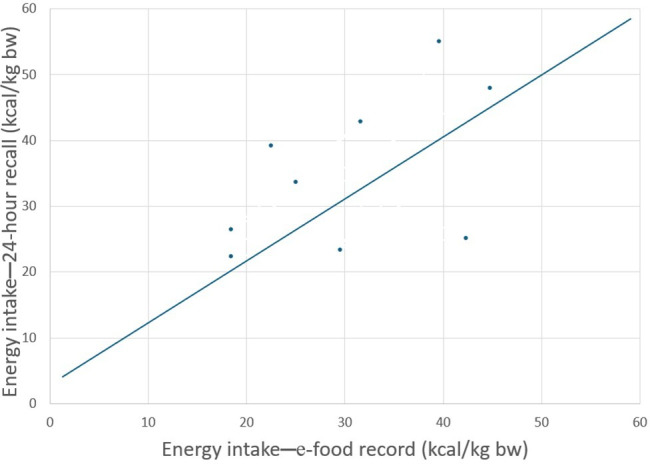
Mean energy intake per participant (n=9) over 3 days, recorded by the e-food record and 24-hour recall. bw: body weight.

The data of 9 participants (group A) were included in the following analysis. The mean energy intake determined with the e-food record was 30.3 (SD 10.2) kcal/kg bw compared to 35.0 (SD 11.0) kcal/kg bw determined with the 24-hour recall. The protein intake determined via the e-food record was 1.2 (SD 0.4) g/kg bw, which was lower than the protein intake determined via the recall at 1.3 (SD 0.4) g/kg bw. Figures4^6^ show the individual participant data of the comparison of the nutritional values for energy, protein, and fluid intake, respectively, according to the e-food record and 24-hour recall. For 7 out of 9 participants, the energy and protein intake determined via the 24-hour recall was higher than that determined via the e-food record. For 6 out of 9 participants, the fluid intake determined via the 24-hour recall was higher than via the e-food record. The mean difference in energy intake between the 24-hour recall and the e-food record was 4.9 (SD 10.2) kcal/kg bw and ranged between −17.2 and 16.7 kcal/kg bw, respectively. For protein intake, the mean difference was 0.1 (SD 0.3) g/kg bw and ranged between −0.5 and 0.4 g of protein/kg bw. Fluid intake differed by 4.9 (SD 9.4) g/kg bw on average and ranged between −13.6 and 18.9 g/kg bw.

**Figure 5.**
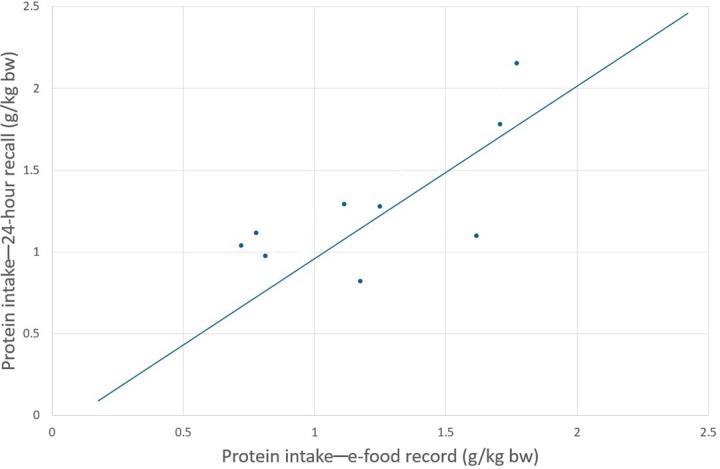
Mean protein intake per participant (n=9) over 3 days, recorded by the e-food record and 24-hour recall. bw: body weight.

**Figure 6.**
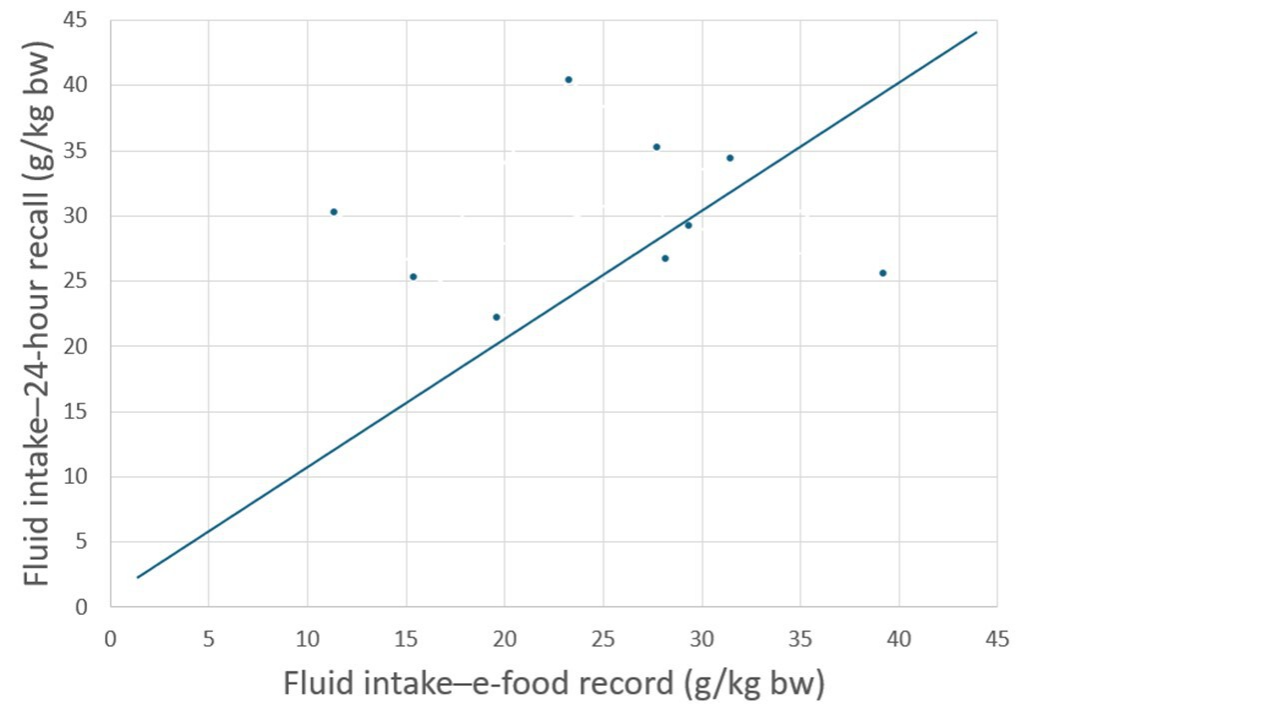
Mean fluid intake per participant (n=9) over 3 days, recorded by the e-food record and 24-hour recall. bw: body weight.

#### Food and Beverage Portion Entries

**Figure 7.**
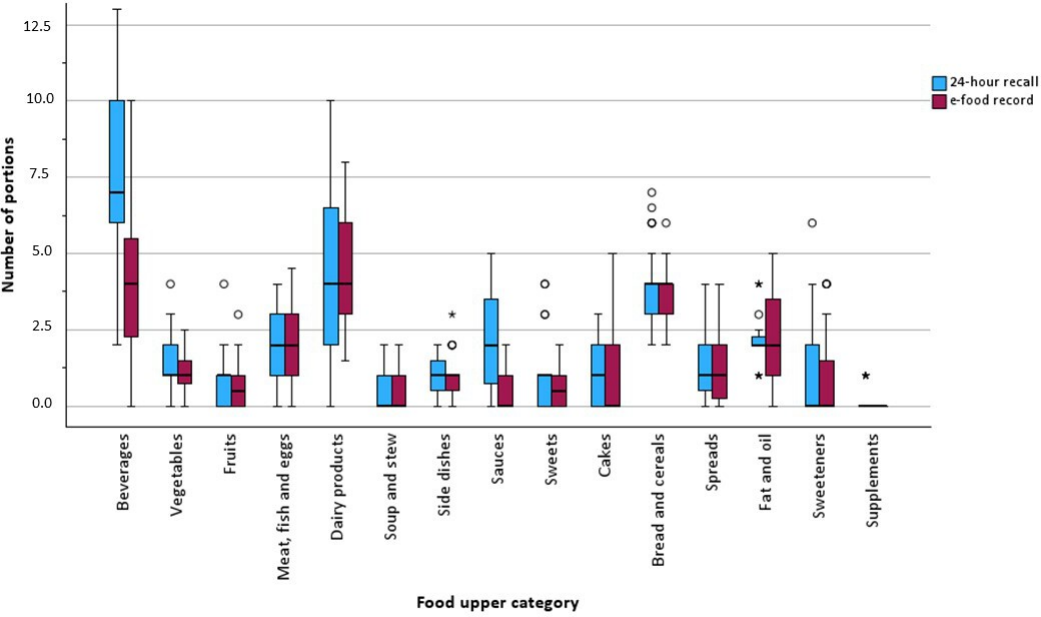
Food and beverage portions per protocol day, recorded by the e-food record and by the 24-hour recall.

Over 3 days and across 9 participants (group A), a total of 625.5 food or beverage portions were recorded by the e-food record and 823.5 food or beverage portions by the 24-hour recall. Per study participant per day, this was an average of 23.9 (SD 5.9) food or beverage portions recorded by the e-food record and 30.8 (SD 6.7) food or beverage portions recorded by the 24-hour recall. On the upper food category level, the median of recorded portions per day was equal for the e-food record and 24-hour recall except for beverages (4, IQR 2-6 vs 7, IQR 6-10.5), fruits (0.5, IQR 0-1 vs 1, IQR 0-1), sauces (0, IQR 0-1 vs 2, IQR 0.5-4), sweets (0.5, IQR 0-1 vs 1, IQR 0-1), and cakes (0, IQR 0-2 vs 1, IQR 0-2), which showed a higher median (IQR) at the 24-hour recall. The results are shown in Figure 7.

**Figure 8.**
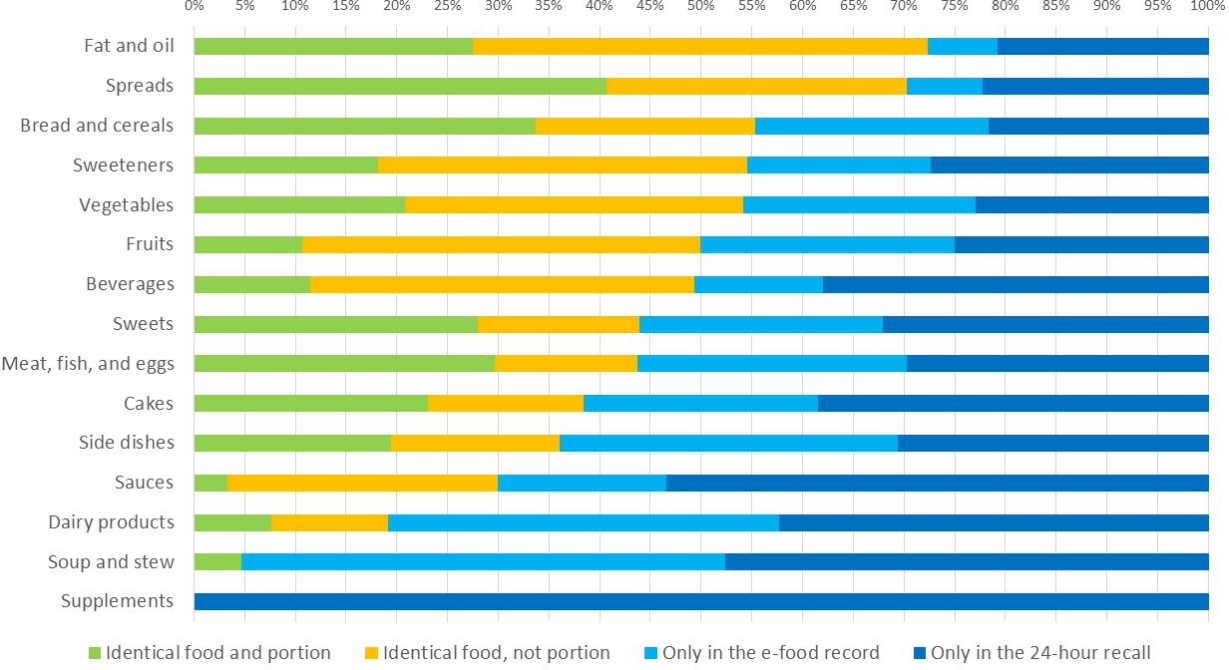
Food and portion entries recorded by the e-food record and 24-hour recall. 100% refers to the total entries of all participants over 3 days.

On the single food level, the portion entries for a corresponding food per day were compared between the e-food record and the 24-hour recall. A consistency was achieved when the same food with the same portion was recorded via the e-food record and the 24-hour recall on the same protocol day. The consistency of the identical portion entries between the e-food record and the 24 h recall was below 30% in 13 out of 15 food categories in relation to all entries in the corresponding food category. The highest correspondence was observed for the food category “spread,” in which 40.7% (n=27) of the foods were recorded in both the e-food record and in the 24-hour recall with the same portion size, followed by 33.8% (n=74) in the food category “bread and cereals” and 27.6% (n=29) in the food category “fat and oil.” In the food category “supplements,” no consistency (0%, n=0) was observed for the portion entries of the e-food record and the 24-hour recall. Further consistency was achieved when the same food, but with a different portion size, was recorded via both the e-food record and the 24-hour recall on the same day. The consistency of food entries with different portion sizes between the e-food record and the 24-hour recall was below 30% in 10 out of 15 food categories in relation to all entries in the corresponding food category. The remaining entries in the food categories were related to foods that were either only recorded via the e-food record or only via the 24-hour recall. The results are shown in Figure 8.

According to the results from Figure 8, a range of food or beverage entries were recorded in only one of the 2 food record methods. In total, 65 different foods or beverages were recorded only via the 24-hour recall by at least 1 participant on at least one of the 3 protocol days. In addition, 48 different foods or beverages were recorded only via the e-food record. Condensed milk, water, and black tea were most often recorded only via the 24-hour recall, and salad and cookies were most often recorded only via the e-food-record. MultimediaAppendices 1  2 represent data for the respective protocol method starting with 3 cases per food and upward.

The explorative analysis of the food and beverage entries with regard to fat content, food variety, and preparation type shows that the same food, but with a different fat level (eg, fruit yogurt with 3.5% fat in dry matter vs fruit yogurt with 10% fat in dry matter), was entered for yogurt, milk and milk drinks, curd, as well as for cold cut and fish. A different type of food (eg, multigrain bread vs wholemeal bread) was entered for cheese, milk and milk drinks, bread, cakes, tea, coffee, fat and oil, vegetables, and fruits. The same dish, but different preparation types, were entered for soup and stew, potato dishes, vegetables, and sauce. Data are represented in Multimedia Appendix 3.

## Discussion

### Principal Results

This pilot study primarily investigated whether an e-food record is a feasible method for documenting nutritional data in older adults undergoing geriatric rehabilitation. The results of this study demonstrate that the e-food record was successfully used by older adults in geriatric rehabilitation to record their food and beverage intake over a period of 3 days. Secondary and exploratory findings are related to usability and the comparison with a 24-hour recall. The e-food record’s usability score (mean 76.0, SD 11.3 points) indicates good acceptability and ease of use. However, the e-food record and the 24-hour recall determined different results in terms of recorded nutrient intake and food portion sizes, indicating possible limitations. These differences should be interpreted with caution, as both methods are subject to measurement errors. While the 24-hour recall may be affected by recall bias and memory limitations, the e-food record may be influenced by input errors or difficulties in the estimation of portion size. These findings suggest that the e-food record is a promising tool for recording food and beverage intake in older adults, but it currently also has some limitations.

### Comparison With Prior Work

With respect to the primary objective of feasibility, this study demonstrated that the target group of patients undergoing geriatric rehabilitation was able to use an e-food record independently during their rehabilitation treatment. A systematic review by Kraaijkamp et al [12] showed that eHealth is feasible, usable, and effective in geriatric rehabilitation. However, none of the 40 included studies were related to nutritional content. A nutrition and exercise e-coach was developed iteratively by Happe et al [13,25] and tested successfully in a piloted application study with a follow-up of 9 weeks among older people during and after geriatric rehabilitation [14]. However, this study was designed for usability testing and user behavior analysis, and the data of the e-food record were not analyzed. A study on community-dwelling older adults with a mean age of 72.2 (range 65-89 y) demonstrated that an e-food record is comparable to a paper-based food record method [26]. During the study, 8 out of 25 participants withdrew prematurely from the study. These results are comparable to the study of Happe et al [14] mentioned above. Here, 8 out of 30 participants dropped out of the study prematurely. In this study, we observed a large difference between the number of patients who were in principle eligible for participation and the number of patients who actually participated (25 out of 224 patients undergoing geriatric rehabilitation).

In contrast, findings related to usability and data comparability should be interpreted as secondary and exploratory, as these aspects were not the primary focus of the study. The usability was rated with a mean score of 76.0 (SD 11.3) points, which reflects a high usability of the e-food record. A score of 82 points is described with the adjective “good” in the evaluation by Bangor et al [23] and is near to the score of 85 from which excellent usability begins. The SUS score is slightly lower than that for a tablet computer−based e-coach to improve nutrition and physical activity in patients undergoing geriatric rehabilitation developed by Happe et al [14] with 78.6 (SD 18.1) points on average. This study was conducted as a 3-day application test that did not contain any increased benefits for the participants (eg, feedback on dietary behavior or dietary recommendations). A higher benefit for the participants and thus a higher willingness to participate could be achieved in future studies by providing feedback on nutritional intake, integrating the e-food record into the nutrition therapy, or extending the study period beyond the rehabilitation program.

### Implications for Research

The demonstrated feasibility of independent use of the e-food record provides an important basis for future research in the field of nutritional assessment in geriatric rehabilitation. The e-food record shows potential as a tool for documenting nutritional data in geriatric rehabilitation, which should be emphasized in future research. In order to exploit the full potential of the e-food record in the future, it should be integrated into daily treatment, ideally as part of a randomized and controlled intervention study. A comparison between the e-food record and the recorded nutritional values should be conducted using established reference methods and a larger number of participants to further investigate the validity and reliability of the e-food record in adequately powered studies. In addition, subsequent studies should investigate which factors contribute to the successful use of the e-food record and which barriers exist to use an e-food record among patients undergoing geriatric rehabilitation. Relevant aspects for further development and optimization of the e-food record were identified in this study by comparing the e-food record with the practical method of the 24-hour recall. The data in Figure 8 indicate that the minority of food and beverage entries (<30 % in 13 out of 15 food categories) are consistent between the e-food record and the 24-hour recall. To estimate portions more precisely, images of food items with the corresponding portions could be displayed on the user interface of the e-food record. In literature, there are also attempts to estimate portions using photo documentation and image recognition [27-29]. Furthermore, the data of Figure 8 indicate a relatively large discrepancy between the foods recorded via the e-food record and the 24-hour recall. A confirmation prompt could be added to the end of a meal entry, for example, to detect incorrect entries by the patients and give them the opportunity to correct them. The results represented in Multimedia Appendix 2 indicate that certain foods such as condensed milk, water, black tea, coffee, cookies, and gravy were partly reported only via the 24-hour recall. This might be due to the fact that the 24-hour recall was queried using a predefined list of foods in food or meal groups that possibly counteracted the forgetting of foods and beverages consumed. A structured query of frequently forgotten or often related foods or beverages such as coffee and condensed milk, bread and spreadable fat, or salad and dressing could prevent forgetting of foods and beverages. The results of Multimedia Appendix 3 indicate that participants may have rated the fat content of foods such as yogurt milk drinks, curd cheese, cold cuts, and fish differently in the e-food record and 24-hour recall. A barcode scanner function or letter recognition of the packaging may increase input accuracy. Within the use of the e-food record, it was possible to select both individual foods and entire dishes. Entire dishes could be omitted. They could be replaced by individual foods. If entire dishes are used as an input option, ingredient queries may increase accuracy of, for example, a stew. However, it is important to balance the benefits of additional functionality against the potential increase in complexity to ensure ease of use for patients undergoing geriatric rehabilitation. The exploratory findings of this study, particularly regarding usability and discrepancies with the 24-hour recall, provide important directions for further development and hypothesis generation.

### Limitations

This study was aimed at serving as a pilot feasibility test and was, therefore, designed as a short-term observation with only a limited number of participants. This could limit the generalizability of the results. In addition, the study was conducted in a single rehabilitation center, which may limit the transferability of the results to other settings or patient populations.

Only a small number of eligible patients participated in the study, and even a smaller number contributed analyzable dietary data. This may introduce substantial selection bias and limited generalizability, as participants who agreed to participate may differ systematically from nonparticipants, for example, in terms of motivation, health status, or technology affinity. Due to ethical constraints, no data on nonparticipation were collected, although the characteristics of this highly burdened population undergoing geriatric rehabilitation likely contributed to the low participation rate. As a result, the findings are based on a highly selected subgroup, and their generalizability to the broader population of patients undergoing geriatric rehabilitation is strongly limited.

A technical malfunction occurred during the study, as the automatic navigation to the current day’s page did not function as intended. This may have affected user interactions and data quality. Feasibility was operationalized primarily based on the dropout rate. However, more detailed indicators of feasibility, such as the level of assistance required during use or the frequency of user errors, were not systematically recorded. This limits a more comprehensive understanding of independent use.

Furthermore, the cognitive status was not systematically recorded within the study. Cognitive impairment may have influenced both the ability to use the e-food record and the accuracy of the 24-hour recall, for example, due to recall bias or difficulties in reporting. However, patients with inability to understand the study content and procedures were excluded from participation, which may partially reduce the impact of severe cognitive impairment in the study sample.

Differences observed between the e-food record and the 24-hour recall could be interpreted with caution, as both methods are subject to different sources of measurement error. The comparison between the two methods was exploratory and not intended as a validation study. Given the small sample size and exploratory design, no firm conclusions regarding the validity or accuracy of either method can be drawn. In addition, analyses related to usability and comparison with the 24-hour recall were exploratory and not powered for definitive conclusions. These results should therefore be interpreted with caution and require confirmation in larger studies.

### Implications for Practice

Effective nutrition therapy is crucial for achieving an optimal nutritional status in patients undergoing geriatric rehabilitation with a reduced nutritional status, as malnutrition is directly linked to physical functionality and rehabilitation outcomes [8]. The e-food record method offers several advantages, including direct access to nutritional information for nutrition therapists. This contributes to the implementation of early and targeted interventions. Traditional food records require manual analysis by nutrition therapists and are often limited to 1-day recalls. With the e-food record method, the patients record their nutritional intake themselves. This reduces personnel resources, which in turn can be used for personalized advice.

By utilizing consumption data, the e-food record can improve nutritional status by providing more intensive and targeted therapy. To date, the detailed data on food and beverage intake over several days are rarely available in clinical practice, although it can enhance counseling. Additionally, nutrition therapists can use the e-food record to monitor the progress of nutrition therapy and introduce new interventions when necessary. This was previously more difficult due to time and financial constraints. This can lead to improved patient outcomes, such as enhanced physical function, reduced hospitalization rates, and better quality of life. Furthermore, an e-food record can facilitate communication between health care professionals, patients, and family members, promoting a more collaborative approach to nutrition therapy. In addition, the use of the e-food record is transferable to patient groups with similar requirements for the use of technology, such as patients in acute geriatric, older patients in neurology, or older people in short-term care facilities. However, given that findings beyond feasibility are exploratory, further validation is required before routine implementation in clinical practice.

### Conclusions

This pilot feasibility study of an e-food record in the population of inpatients undergoing geriatric rehabilitation is, to our knowledge, the first of its kind and demonstrated, in general, that patients undergoing geriatric rehabilitation were able to independently record their food and beverage intake using the e-food record over 3 days. The results suggest that app-based food recording can be an integral part of nutrition therapy in the future to improve the medical care of patients undergoing geriatric rehabilitation. However, the findings of this study show that the e-food record and the 24-hour recall provide different results in terms of nutrient intake and food portion sizes. Further research with studies involving larger numbers of participants is needed to draw conclusions regarding data accuracy or validity of the e-food record.

## Supplementary material

10.2196/84653 Multimedia Appendix 1 Food recorded only via the 24-hour recall.

10.2196/84653 Multimedia Appendix 2 Food recorded only via the e-food record.

10.2196/84653 Multimedia Appendix 3 Food entries according to fat content, food varieties, and preparation type.

## Abbreviations

bw: body weight
SUS: System Usability Scale
