# Supplementary figures and images for: A Tablet Computer–Based Food Record for the Self-Assessment of Nutritional Intake in Patients Undergoing Geriatric Rehabilitation: Prospective Pilot Feasibility Study
Source: JMIR Aging. 2026 Jul 9;9:e84653. doi: 10.2196/84653 (PMC13348802; doi:10.2196/84653)

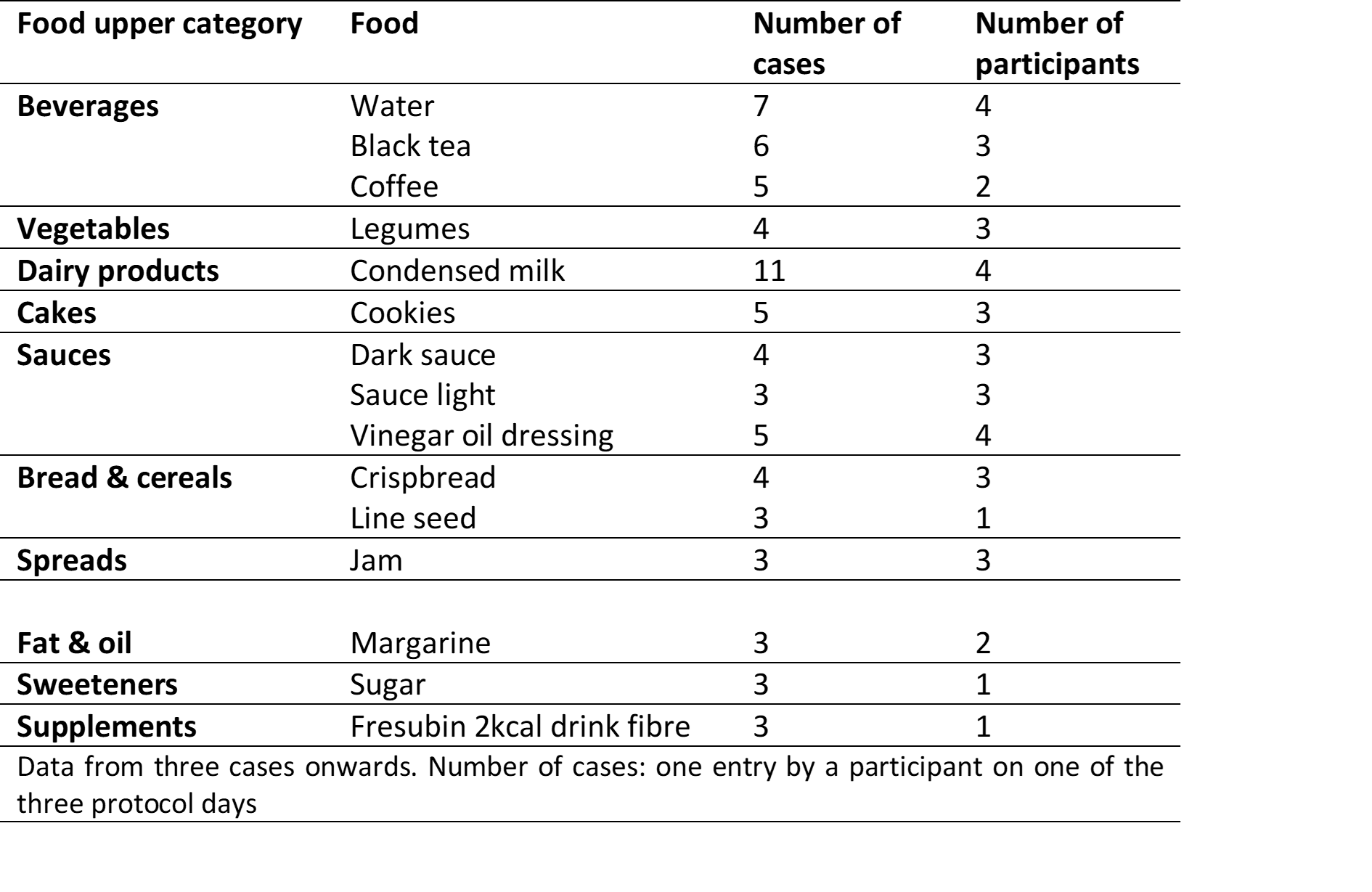

Supplement: Multimedia Appendix 1 [file aging-v9-e84653-s001.png]

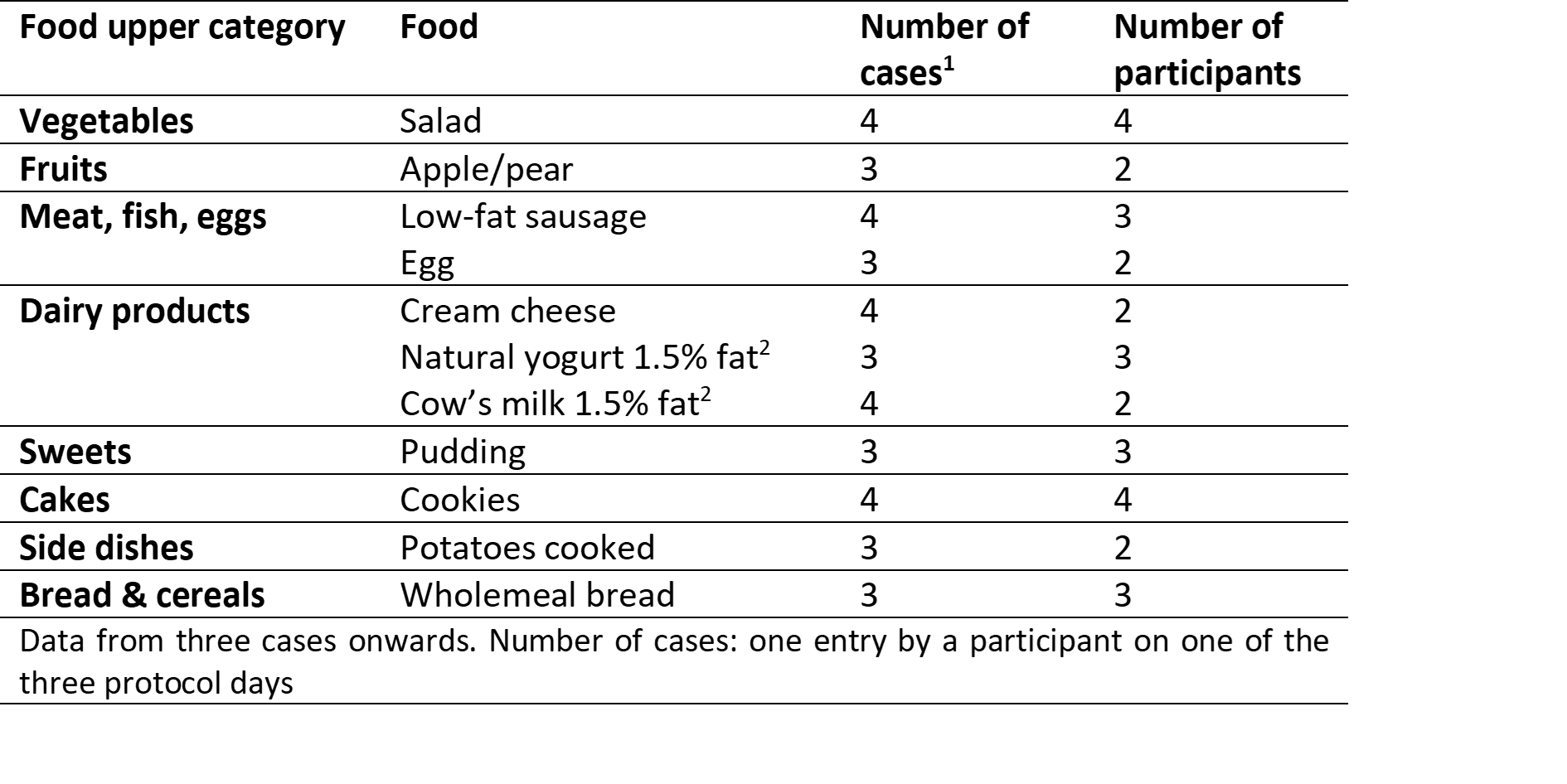

Supplement: Multimedia Appendix 2 [file aging-v9-e84653-s002.png]

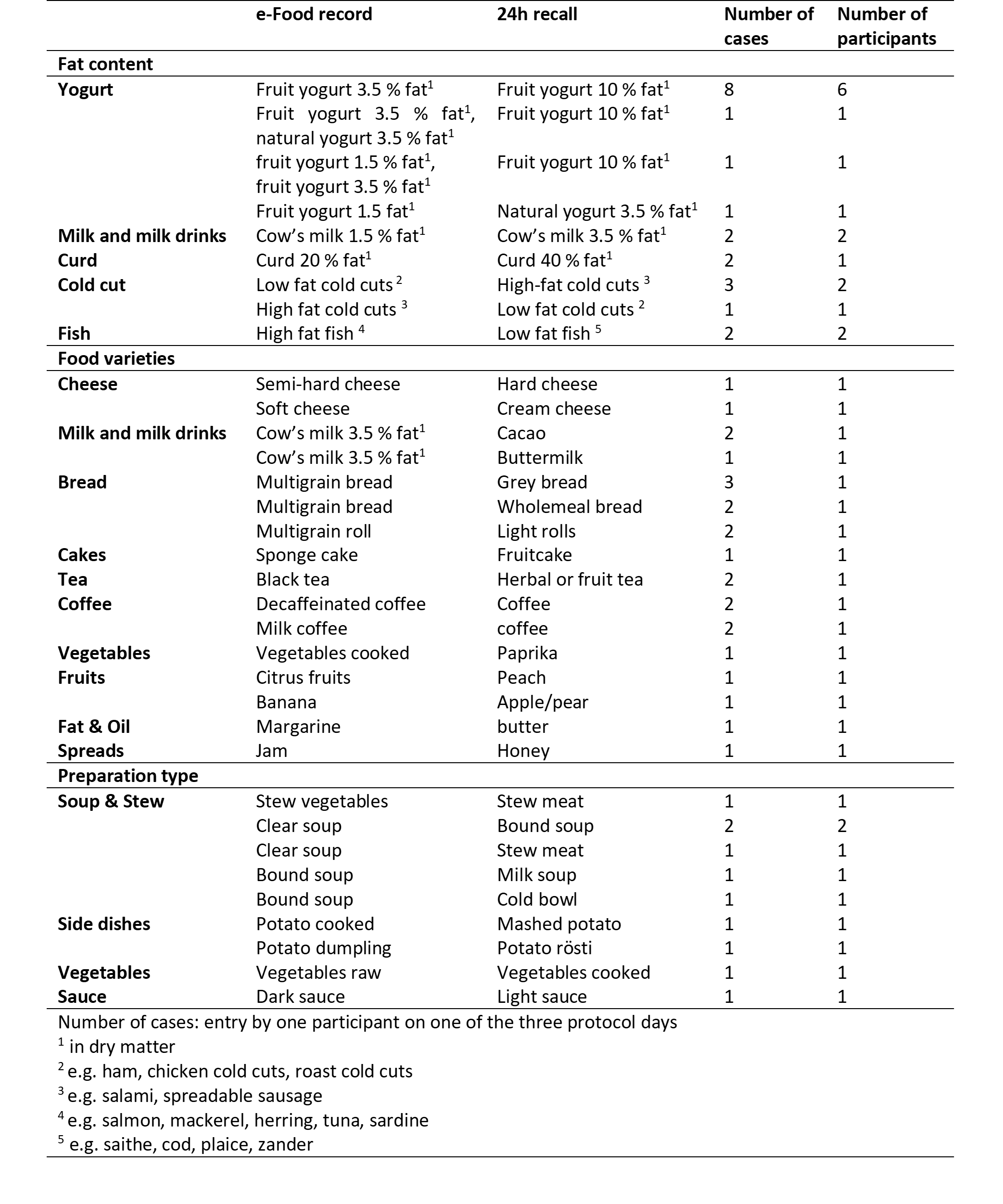

Supplement: Multimedia Appendix 3 [file aging-v9-e84653-s003.png]
